# Alcohol and breast cancer risk among Asian-American women in Los Angeles County

**DOI:** 10.1186/bcr3363

**Published:** 2012-11-27

**Authors:** Anna H Wu, Cheryl Vigen, Pedram Razavi, Chiu-Chen Tseng, Frank Z Stancyzk

**Affiliations:** 1 Department of Preventive Medicine, University of Southern California Keck School of Medicine, 1441 Eastlake Avenue, Los Angeles, CA 90089, USA; 2 Division of Occupational Science and Occupational Therapy, University of Southern California Keck School of Medicine, 1540 Alcazar Street, CHP-133, Los Angeles, CA 90089, USA; 3 Department of Medicine, University of Southern California Keck School of Medicine, 1200 N State Street, Los Angeles CA 90089, USA; 4 Department of Obstetrics and Gynecology, University of Southern California Keck School of Medicine, 201 Livingston Research Building, Los Angeles, CA 90089, USA

## Abstract

**Introduction:**

The role of alcohol and breast cancer risk in Asians has not been well studied. Recent studies suggest that even moderate alcohol intake may be associated with an increase in breast cancer risk, and this may be particularly relevant as alcohol intake is traditionally low among Asians.

**Methods:**

We investigated the association between lifetime alcohol intake (including frequency, quantity, duration, timing, and beverage type) and breast cancer in a population-based case-control study of 2,229 Asian Americans diagnosed with incident breast cancer and 2,002 matched control women in Los Angeles County. Additionally, we examined the relation between current alcohol intake and serum concentrations of sex-hormones and growth factors in a subset of postmenopausal control women.

**Results:**

Regular lifetime alcohol intake was significantly higher in US-born than non-US-born Asian Americans (*P *< 0.001) and almost twice as common in Japanese- than in Chinese- and Filipino-Americans (*P *< 0.001). Breast cancer risk increased with increasing alcohol intake among US-born Asian Americans; the odds ratios (ORs) per 5 grams per day and per 10 years of drinking were 1.21 (95% confidence interval (CI) 1.00 to 1.45) and 1.12 (95% CI, 0.98 to 1.28), respectively. Regular alcohol intake was a significant risk factor for Japanese-, but not for Chinese- and Filipino-Americans. Current consumers compared with nondrinkers showed lower concentrations of insulin-like growth factor binding protein 3 (*P *= 0.03) and nonsignificantly higher concentrations of estrone and androgens.

**Conclusions:**

Regular lifetime alcohol intake is a significant breast cancer risk factor in US-born Asian Americans and Japanese Americans, emphasizing the importance of this modifiable lifestyle factor in traditionally low-risk populations.

## Introduction

High consumption of alcohol has been associated consistently with an increased risk of breast cancer in studies conducted primarily among women of European ancestry [1]. The role of alcohol and breast cancer development in Asia and in Asian American women has been less studied, and the results are not consistent [2,3]. Reasons for the inconsistent results in Asians may be related, in part, to the traditionally low alcohol intake in this population, the variable definition of alcohol intake in different studies, and often, the limitation of the assessment to current alcohol intake. The Nurses' Health Study, which considered cumulative alcohol intake, reported significant increased breast cancer risk (15%, from 6% to 24%), even at modest levels of alcohol intake (5 to 9.9 g per day or three to six drinks per week) [4], emphasizing the importance of considering lifetime alcohol intake.

We recently completed a large population-based case-control study of breast cancer among Asian-American women in Los Angeles County (LAC) in which detailed information on lifetime alcohol intake (frequency, amount, duration, timing, types of alcoholic beverages) was collected. We investigated the relation between alcohol intake and breast cancer risk separately by Asian ethnicity (Chinese, Japanese, Filipino), nativity (US born, non-US born), and by Asian ethnicity and nativity. We also examined the interrelations between alcohol intake, smoking status, body size, use of hormone therapy (in postmenopausal women), and various dietary factors on breast cancer risk in LAC Asian-American women.

## Materials and methods

### Study design and population

The study population and methods used in this population-based case-control study have been described previously [5]. In brief, breast cancer patients were identified by the LAC Cancer Surveillance Program (CSP), the population-based cancer registry covering Los Angeles, a member of the statewide California Cancer Registry, and the National Cancer Institute's Surveillance, Epidemiology, and End Results (SEER) program. Case patients were identified as Chinese, Japanese, or Filipino between the ages of 25 and 74 years, inclusive, at the time of diagnosis of an incident breast cancer. Cases were diagnosed between 1995 and 2001 and between 2003 and 2006. In total, we identified 3,797 eligible case patients (1,496 Chinese, 865 Japanese, 1,436 Filipino) and interviewed 2,303 cases (929 Chinese, 547 Japanese, 827 Filipino). Among those who did not participate, 869 declined to be interviewed (375 Chinese, 222 Japanese, 272 Filipino), 77 had died (17 Chinese, 24 Japanese, 36 Filipino), and 548 could not be located (175 Chinese, 72 Japanese, 301 Filipinos). The 2,035 control subjects (923 Chinese, 518 Japanese, 594 Filipino) were selected from the neighborhoods where the case patients resided at the time of diagnosis. A well-established algorithm was used to identify neighborhood controls that we have used in numerous case-control studies [6,7]. We initially defined a specified sequence of houses to be visited in the neighborhoods where index cases lived at the time of diagnosis. We then sought to interview the first eligible resident in the sequence. If the first eligible control subject refused to participate, the second eligible one in the sequence was asked, and so on. Letters were left when no one was home, and follow-up was by mail and telephone. Controls were sought to frequency-match to the cases on specific Asian ethnicities and 5-year age groups. On average, a suitable control was identified after visiting a median of 60 households. Of the controls interviewed, 64% were the first-identified eligible control, 18% were the second-identified eligible control, and 18% were the third or later eligible control.

### Data collection

Cases and controls were interviewed in person by trained multilingual interviewers by using a standardized, structured questionnaire. In brief, the questionnaire elicited information on race/ethnicity, migration history, education, family history of cancer, menstrual and reproductive histories, use of exogenous hormones, smoking history, lifetime physical-activity patterns, birth weight, and height and weight history. We used two separate measures to assess alcohol intake. In the beginning of the interview, by using a quantitative food-frequency questionnaire (FFQ) that was modeled after the validated diet instrument used in the Multiethnic Cohort Study in Hawaii and Los Angeles [8], we asked women about usual intake of specific foods/food groups and beverages during the year before cancer diagnosis (for cases) or during the past year (for controls). Separate questions were asked regarding intake of beer, wine, and hard liquor. The FFQ recorded frequency of intake (never or < 1/month, 1 time per month, 2 to 3 times per month, 1 time per week, 2 to 3 times per week, 4 to 6 times per week, once per day, 2 or more times per day) for each food/beverage and the usual amounts of intake for at least monthly consumption. A second measure of alcohol intake was based on lifetime-history assessment; these questions were asked toward the end of the interview. Specifically, we asked if they ever drank alcoholic beverages on a regular basis, defined as at least once a week for 6 months or longer or at least 26 drinks per year before the reference date. For regular drinkers, we asked the age they first started drinking alcoholic beverages regularly, the age they last drank regularly, the total number of years they drank regularly, and the average number of drinks they consumed per day or per week when they drank regularly. These questions were asked separately for intake of beer, wine, sake, and hard liquor. Subjects were told that one drink is equivalent to one bottle or 12-oz can of beer, one glass (or 4 oz) of wine, 1-oz cup sake, or one cocktail or mixed drink of hard liquor (1.5-oz shot). Information on tumor characteristics including estrogen receptor (ER) and progesterone receptor (PR) status was routinely collected by the LAC-CSP as part of SEER study protocol.

Variables on body size, use of menopausal hormones, diabetes, selected dietary factors, and dietary pattern have been reported in the first group of study participants (1,384 cases, 1,225 controls) [9-11]. The relation between birth weight and breast cancer risk has been reported in all participants [5]. The study was approved by the Institutional Review Board of the Keck School of Medicine at the University of Southern California. Informed consent was obtained from each case and control before her interview.

### Measurement of biomarkers in control women

Blood specimens were collected from interviewed breast cancer cases and control women at the end of the interview. We conducted a cross-sectional study among a subset of postmenopausal control women in which sex hormones and growth factors were measured. Methods used to measure these biomarkers have been published in our previous studies [10-12]. In brief, we used commercially available enzyme-linked immunosorbent assay (ELISA) kits to measure insulin-like growth factor (IGF)-1, IGF-binding protein (BP)-3, sex-hormone-binding globulin (SHBG), adiponectin, and dehydroepiandrosterone sulfate (DHEAS). Serum levels of estradiol (E2), estrone (E1), testosterone (T), and androstenedione (adione) were measured with previously described radioimmunoassay methods [13].

### Statistical analysis

In total, 2,229 cases and 2,002 controls with complete information on lifetime alcohol intake and the covariates included for adjustment were included in this study (74 cases and 33 controls with missing data on alcohol intake or covariates were excluded). For drinkers, the number of grams of ethanol consumed per day was estimated by multiplying the number of drinks of each specific type of alcohol consumed per week by the appropriate number of grams of ethanol per drink, and then summing across the type of alcohol and dividing by seven. Ethanol values attributed to a 12-oz serving of beer, 4-oz serving of wine, 1-oz serving of sake, and 1.5-oz serving of hard liquor were 13, 11, 4, and 14 grams, respectively.

We calculated odds ratios (ORs; relative risk estimates) and their corresponding 95% confidence intervals (CIs) and *P *values by conditional logistic regression methods, with matched sets defined jointly by reference age (< 39, 40 to 44, 45 to 49, 50 to 54, 55 to 59, 60 to 64, 65 to 69, and 70+ years), and specific Asian ethnicity (Chinese, Japanese, Filipino). All regression models included the following covariates: birthplace and years of residence in the United States among non-US born (> 20 years, 11 to 20 years, ≤ 10 years), education (less than high school, high school, some college, college graduate), interviewer, age at menarche (< 12, 12 to 13, or ≥ 14 years), parity (0, one, two, three, or four or more births), current body mass index (BMI (kg/m^2^); quartiles of controls), years of regular (that is, at least 1 hour per week) recreational physical activity (< 5, 5 to 9, 10 to 19, or ≥ 20 years), total calories (continuous), intake of soy (quartiles), green tea (0, < 120 ml/day, or ≥ 120 ml/day), and black tea (0, < 120 ml/day, or ≥ 120 ml/day), menopausal status (premenopausal, natural menopause, bilateral oophorectomy, simple hysterectomy, or "hormone therapy menopause" defined as starting hormone therapy before periods had stopped), age at menopause (≤ 44, 45 to 49, 50 to 54, or 55+ years), and family history of breast cancer. We conducted case-control comparisons for all subjects combined, and separately by Asian ethnicity (Chinese, Japanese, Filipino), nativity (US born, non-US born), and by the six Asian ethnic/nativity groups, and menopausal status. To examine the potential effect modification of smoking, BMI, soy intake, folate intake, and use of menopausal hormones on association with alcohol and breast cancer, we separately constructed interaction terms between these factors and alcohol intake and used likelihood-ratio tests to assess the significance of these interaction terms. We used analysis of variance (ANOVA) and analysis of covariance (ANCOVA) to assess the relations between alcohol intake and serum hormone levels and other biomarkers in a subgroup of control women after adjusting for age, Asian ethnicity, migration history, education, age at menarche, parity, and current BMI. Hormone and other biomarker measurements were transformed logarithmically to achieve approximate normal distributions for statistical analysis. We evaluated whether the geometric mean of hormone and other biomarker levels differed by current drinking status. *P *values less than 0.05 are considered statistically significant, and all *P *values quoted are two-sided. All analyses were performed by using the EPILOG statistical software system (version 1.01s; Pasadena, CA, USA) and the SAS statistical software (version 8.0; SAS Institute, Cary, NC, USA).

## Results

Table 1 (top half) shows current alcohol use (defined as at least 12 times per year) during the 12 months before interview for control women by Asian ethnicity (Japanese, Chinese, Filipino), nativity (US born, non-US born), and each Asian ethnicity by US born or non-US born. The percentage of current drinkers differed between the six Asian ethnic-nativity groups (*P *< 0.0001). The percentage of current drinkers was highest in Japanese women (33.3%), intermediate in Chinese (21.9%), and lowest in Filipino (18.3%) (*P *< 0.0001). The percentage of current drinkers was also higher in US-born Asians (29.2%) than in non-US-born Asians (21.8%) (*P *= 0.0005). However, among current drinkers, no significant differences were found in the frequency of drinking (times per year) or average amount of alcohol consumed per day by Asian ethnicity, by nativity, or by Asian ethnicity and nativity combined. Lifetime alcohol-intake (defined as at least weekly for 6 months or 26 times per year) patterns also differed between the six Asian ethnic-nativity groups (*P *< 0.0001). Regular lifetime alcohol drinking was almost twice as common in Japanese (26.7%) compared with Chinese (13.7%) and Filipino (14.1%) control women (*P *< 0.0001). The percentage of regular lifetime drinking was higher in US-born Asians (24.2%) compared with non-US-born Asians (14.5%) (*P *< 0.0001). However, among regular drinkers, no significant differences were found in the amount and duration of drinking between the three Asian groups or between US-born and non-US-born Asian Americans.

**Table 1:** Current and lifetime alcohol intake in control women, by Asian ethnicity, and by nativity

	Asian ethnicity			Nativity		Asian ethnicity and nativity					
						Japanese		Chinese		Filipino	
	**Japanese** ***n *= 510**	**Chinese** ***n *= 903**	**Filipino** ***n *= 587**	**US born** ***n *= 537**	**Non-US** ***n *= 1,463**	**US born** ***n *= 378**	**Non-US** ***n *= 132**	**US born** ***n *= 131**	**Non-US** ***n *= 772**	**US born** ***n *= 28**	**Non-US** ***n *= 559**

Current alcohol (≥ 12 times/year) (12 months before diagnosis/interview)											

No (%)	340 (66.7)	705 (78.1)	479 (81.7)	380 (70.8)	1,144 (78.2)	266 (70.4)	74 (56.1)	95 (72.5)	610 (79.0)	19 (67.9)	460 (82.3)
Yes (%)	170 (33.3)	198 (21.9)	108 (18.3)	157 (29.2)	319 (21.8)	112 (29.6)	58 (43.9)	36 (27.5)	162 (21.0)	9 (32.1)	99 (17.7)
*P*			< 0.0001		0.0005		0.003		0.10		0.54
Adj number of times/year^a^	136.9	106.6	120.1	115.7	126.7	127.8	149.8	117.5	108.1	96.8	127.1
*P*			0.16		0.47		0.71		0.82		0.35
Adj alcohol g/day^a^	5.1	3.8	4.1	4.2	4.5	4.5	6.0	4.9	3.7	3.7	4.3
*P*			0.21		0.61		0.41		0.35		0.43

Lifetime alcohol intake (at least once per week for 6 months or 26 times per year)											

No (%)	374 (73.3)	779 (86.3)	504 (85.9)	407 (75.8)	1,252 (85.5)	282 (74.6)	92 (69.7)	106 (80.9)	673 (87.2)	19 (67.8)	485 (86.7)
Former (%)	56 (10.4)	36 (4.0)	32 (5.5)	59 (11.0)	72 (4.9)	47 (12.4)	9 (6.8)	8 (6.1)	28 (3.6)	4 (14.3)	31 (5.6)
Current (%)	80 (16.3)	88 (9.7)	51 (8.6)	71 (13.2)	141 (9.6)	49 (13.0)	31 (23.5)	17 (13.0)	71 (9.2)	5 (17.9)	43 (8.1)
*P*			< 0.0001		< 0.0001		0.007		0.16		0.02
Adj alcohol years^a^	15.5	14.4	11.7	13.6	14.0	14.6	16.7	13.3	14.8	18.7	10.9
			0.17		0.84		0.58		0.32		0.04
Adj alcohol g/day^a^	5.7	6.1	8.7	7.7	5.9	6.6	4.7	6.4	5.4	10.8	7.6
			0.15		0.21		0.33		0.84		0.62

^a^Asian ethnicity: Adjustment included age, birthplace, and years of residence in the United States among non-US born, education, and interviewer. Nativity: Adjustment included age, Asian ethnicity, years of residence in the United States among non-US born, education, and interviewer. Asian Ethnicity and Nativity combined: Adjustment included age, years in the US for non-US born, education, and interviewer.

Current alcohol intake was unrelated to breast cancer risk in all subjects combined (adjusted OR, 0.87; 95% CI, 0.74 to 1.03). However, risk patterns differed by Asian ethnicity and nativity (Table 2). In Chinese (US-born and non-US-born) and non-US-born Filipino women, breast cancer risk was < 1.0 among current drinkers; the trends with the amount of current alcohol intake were borderline statistically significant. Breast cancer risk in non-US-born Japanese was unrelated to current alcohol intake. However, among US-born Japanese, breast cancer risk was higher among current drinkers (adjusted OR, 1.42; 95% CI, 0.98 to 2.05; *P *= 0.062) and increased with increasing frequency of drinking (*P *trend = 0.053). The risk per 5 g/day of intake was 1.32 (95% CI, 1.01 to1.73; *P *trend = 0.044). In US-born Filipino women, breast cancer risk was nonsignificantly elevated in association with current alcohol intake (adjusted OR per 5 g/day = 2.11; 95% CI, 0.78 to 5.71; *P *trend = 0.14).

**Table 2:** Current alcohol intake (at least 12 times per year) assessed by quantitative food-frequency questionnaire and risk of breast cancer by Asian ethnicity and birthplace

	Japanese				Chinese				Filipino			
Cases/controls	US born 364/378		Non-US born 177/132		US born 90/131		Non-US born 808/772		US born 22/28		Non-US born 764/559	
	**Ca/Co**	**OR**^a^	**Ca/Co**	**OR**^a^	**Ca/Co**	**OR**^a^	**Ca/Co**	**OR**^a^	**Ca/Co**	**OR**^a^	**Ca/Co**	**OR**^a^
No	248/266	1.00	112/74	1.00	73/95	1.00	685/610	1.00	11/19	1.00	658/460	1.0
Yes	116/112	1.42	65/58	0.78	17/36	0.31	123/162	0.72	11/9	2.93	106/99	0.79
*P *value		0.062		0.41		0.015		0.022		0.11		0.18
None	248/266	1.00	112/74	1.00	73/95	1.00	685/610	1.00	11/19	1.00	659/460	1.00
> 0 to ≤ 4/week	89/90	1.36	45/45	0.73	14/30	0.29	96/142	0.63	10/9	2.67	91/79	0.87
> 4/week	27/22	1.67	20/13	0.97	3/6	0.43	27/20	1.36	1/0		14/20	0.43
*P *trend		0.053		0.61		0.035		0.16		0.27		0.05
*P *interaction				0.49				0.73				0.08
Current amount (g/day)												
None	248/266	1.00	112/74	1.00	73/95	1.00	685/610	1.00	11/19	1.00	659/460	1.00
> 0 to ≤ 5.0	85/85	1.33	41/44	0.62	10/27	0.22	95/132	0.68	9/7	2.95	86/74	0.90
> 5	31/27	1.73	24/14	1.32	7/9	0.63	28/30	0.92	2/2	2.88	20/25	0.49
Per 5 g/day		1.32		0.99		0.55		0.82		2.11		0.78
95% CI		1.01-1.73		0.66-1.48		0.29-1.05		0.66-1.02		0.78-5.71		0.60-1.02
*P *trend		0.044		0.96		0.07		0.07		0.14		0.07
*P *interaction				0.42				0.37				0.22

Summary by Asian ethnicity	Japanese				Chinese				Filipino			
By 5 g/day	1.17 (0.99 to 1.39); *P *trend = 0.073				0.83 (0.70 to 0.99); *P *trend = 0.032				0.84 (0.68 to 1.03); *P *trend = 0.096			
*P *interaction	0.016											
Summary by nativity	US Born				Non-US born							
By 5 g/day	1.16 (0.97 to 1.39); *P *trend = 0.073				0.82 (0.73 to 0.93); *P *trend = 0.002							
*P *interaction	0.027											

^a^Adjusted for age, years of residence in the United States among non-US born, education, interviewer, age at menarche, menopausal status, age at menopause, current BMI, parity, total calories, family history of breast cancer and physical activity, and intake of soy and green tea.

Results on breast cancer risks in relation to lifetime alcohol intake are shown in Table 3. Lifetime alcohol intake was not significantly associated with risk in US-born Chinese, non-US-born Chinese, and non-US-born Filipinos, although most of the risk estimates were < 1.0. In contrast, breast cancer risk in US-born Filipinas and Japanese Americans (US-born and non-US-born) tended to increase with increasing lifetime alcohol intake. Specifically, in US-born Japanese Americans, risk increased with increasing amount (adjusted OR per 5 g was 1.24; 95% CI, 1.01 to 1.53; P trend = 0.038) and with increasing duration of intake (adjusted OR per 10 years was 1.18; 95% CI, 1.01 to 1.38), although the finding with duration was no longer statistically significantly after additionally adjusting for amount of alcohol intake. US-born Japanese women who consumed > 5 g/day and > 10 years of intake showed an OR of 1.65 (95% CI, 0.86 to 3.15); risk was significantly higher with cumulative lifetime alcohol intake (*P *trend = 0.022). Breast cancer risks in non-US-born Japanese and US-born Filipinos were similarly increased in association with lifetime alcohol intake; the OR per 5 g/day of alcohol intake was 1.23 (0.86 to 1.77) and 1.90 (95% CI, 0.65 to 5.49), respectively.

**Table 3:** Lifetime alcohol intake (at least once per week for 6 months or 26 times per year) and risk of breast cancer by Asian ethnicity and birthplace

	Japanese				Chinese				Filipino			
	US born		Non-US born		US born		Non-US born		US born		Non-US born	
	Ca/Co	**OR**^a^	Ca/Co	**OR**^a^	Ca/Co	**OR**^a^	Ca/Co	**OR**^a^	Ca/Co	**OR**^a^	Ca/Co	**OR**^a^
Lifetime alcohol												
Never	258/282	1.00	121/92	1.00	75/106	1.00	721/673	1.00	13/19	1.00	698/485	1.00
Ever	107/96	1.51	58/41	1.36	16/25	0.86	87/100	0.84	9/9	2.33	66/74	0.66
*P *(1 df)		0.035		0.33		0.75		0.23		0.41		0.053
Never	258/282	1.00	121/92	1.00	75/106	1.00	721/673	1.00	13/19	1.00	698/485	1.00
Former	46/47	1.14	20/9	2.40	7/8	1.43	22/32	0.62	6/4	2.93	30/31	0.91
Current	61/49	1.91	38/32	1.15	9/17	0.68	65/68	0.95	3/5	1.62	36/43	0.53
*P *(2 df)		0.032		0.26		0.70		0.29		0.42		0.057
Amount of alcohol (g/day)												
No	258/282	1.00	121/92	1.00	75/106	1.00	721/673	1.00	13/19	1.00	698/485	1.00
> 0 to ≤ 5.0	64/59	1.43	39/30	1.26	11/21	0.80	56/70	0.80	6/7	2.11	41/47	0.67
> 5 to ≤ 10	18/19	1.52	8/6	1.40	3/2	1.21	15/13	1.14	3/2	3.23	14/12	0.84
> 10	25/18	1.81	11/5	1.93	2/2	0.88	16/17	0.79	----	-----	11/15	0.50
Per 5 g/day		1.24		1.23		0.95		0.92		1.90		0.80
*P *trend		0.038		0.26		0.87		0.43		0.24		0.059
Years of drinking (no adjustment for amount of drinking)												
> 0 to ≤ 5	31/30	1.37	15/10	1.44	4/7	1.93	34/36	0.99	5/3	3.80	22/38	0.41
> 5-10	19/20	1.53	12/9	1.37	2/8	0.63	20/16	1.25	1/1	1.42	14/10	1.17
> 10 years	57/46	1.60	31/22	1.33	10/10	0.69	33/48	0.61	3/5	1.63	30/26	0.85
Per 10 years		1.18		1.11		0.89		0.89		1.22		0.92
*P *trend		0.038		0.41		0.54		0.13		0.52		0.36
Years of drinking, adjusted for amount of drinking (g/day)												
> 0 to ≤ 5		1.22		1.14		1.42		0.92		3.19		0.48
> 5-10		1.32		0.97		0.46		1.14		1.01		1.29
> 10 years		1.34		0.95		0.44		0.56		1.40		0.99
Per 10 years		1.09		0.97		0.70		0.83		0.97		1.17
*P *trend		0.54		0.88		0.37		0.14		0.94		0.34
Amount (g/day) and years of drinking												
No	258/282	1.00	121/92	1.00	75/106	1.00	721/673	1.00	13/19	1.00	698/485	1.00
≤ 5.0 and ≤ 10 years	36/38	1.36	20/14	1.48	5/14	1.14	35/37	0.98	4/2	5.18	25/34	0.58
≤ 5.0 and > 10 years	28/21	1.54	19/16	1.10	6/7	0.51	21/33	0.60	2/5	1.00	16/13	1.10
> 5.0 and ≤ 10 years	14/12	1.68	7/5	1.36	1/1	1.04	19/15	1.30	2/2	3.40	11/14	0.61
> 5.0 and > 10 years	29/25	1.65	12/6	2.10	4/3		12/15	0.62	1/0		14/13	0.74
*P *trend		0.041		0.29		0.74		0.28		0.40		0.21
Lifetime intake												
0	258/282	1.00	121/92	1.00	75/106	1.00	721/673	1.00	13/19	1.00	698/485	1.00
≤ 7.6 kg	43/43	1.28	26/20	1.40	9/16	1.08	31/52	1.02	4/3	3.49	35/40	0.71
> 7.6 kg	64/53	1.71	32/21	1.37	7/9	0.64	56/48	0.66	5/6	1.84	31/34	0.68
*P *trend		0.022		0.36		0.61		0.15		0.38		0.11

Summary by Asian ethnicity	Japanese				Chinese				Filipino			
By 5 g/day	1.21 (1.02-1.43), *P *trend = 0.032				0.95 (0.79-1.14), *P *trend = 0.57				0.85 (0.68-1.07), *P *trend = 0.16			
*P *interaction	0.011											
Summary by nativity	US born				Non-US born							
By 5 g/day	1.21 (1.00-1.45), *P *trend = 0.048				0.91 (0.80-1.04), *P *trend = 0.18							
*P *interaction	0.12											

^a^Adjusted for age, years of residence in the US among non-US born, education, interviewer, age at menarche, menopausal status, age at menopause, current BMI, parity, total calories, family history of breast cancer and physical activity, intake of soy, and green tea.

We also investigated risk patterns by nativity, but by combining across Japanese, Chinese, and Filipino women. In US-born women, the adjusted OR per 5 g/day of alcohol intake was 1.21 (95% CI, 1.00 to 1.45; *P *trend = 0.048), whereas the corresponding OR was 0.91 (95% CI, 0.80 to 1.04) in non-US-born women (*P *interaction = 0.12). The risk estimates did not differ significantly within each of the three Asian ethnic groups by nativity. The OR per 5 g/day of lifetime alcohol intake in Japanese, Chinese, and Filipino women was 1.21 (95% CI, 1.02 to 1.43), 0.95 (95% CI, 0.79 to 1.14), and 0.85 (95% CI, 0.68 to 1.07), respectively (Table 3, bottom). In Japanese women and in US-born Asian Americans, all types of alcoholic beverages (beer, wine, sake, and hard liquor) were implicated (data not shown).

We investigated the relation between lifetime alcohol intake and breast cancer risk in Japanese Americans by various potential modifiers, including smoking (never versus ever), intake of soy and folate, BMI, and use of menopausal hormones (among postmenopausal women) (Table 4). The increased risk associated with lifetime alcohol intake was statistically significant among Japanese who were ever smokers but not among Japanese who were never smokers; the difference in association by smoking status was statistically significant (*P *= 0.017). None of the other factors significantly modified the alcohol/breast cancer association in Japanese Americans. Results were similar when we investigated the alcohol/breast cancer associations by potential modifiers in US-born Asian Americans (data not shown).

**Table 4:** Lifetime alcohol intake (g/day) and breast cancer risk in Japanese Americans, stratified by smoking, intake of soy and folate, body mass index (BMI), and use of menopausal hormones (postmenopausal women)

Alcohol (g/day)	Cases/control	**Adjusted OR**^a ^**(95% CI)**	Cases/control	**Adjusted OR**^a ^**(95% CI)**	*P*_interaction _
	Never smoked		Ever smoked		
No	273/240	1.00	106/134	1.00	
> 0 to ≤ 5.0	49/46	0.87 (0.52-1.43)	54/43	2.09 (1.16-3.77)	
> 5	23/20	1.33 (0.65-2.73)	39/28	2.46 (1.26-4.80)	
Per 5 g/day		1.06 (0.77-1.44)		1.64 (1.18-2.26)	
*P *trend		0.73		0.003	0.017

	Low soy intake^b^		High soy intake^b^		
No	126/105	1.00	253/269	1.00	
> 0 to ≤ 5.0	31/23	1.39 (0.65-2.94)	72/66	1.18 (0.77-1.80)	
> 5	19/11	1.01 (0.37-2.72)	43/37	1.49 (0.88-2.55)	
Per 5 g/day		1.10 (0.70-1.71)		1.21 (0.95-1.55)	
*P *trend		0.69		0.13	0.96

	≤ Median folate (≤ 190 mg/1,000 Kcal)^c^		> Median folate (> 190 mg/1,000 Kcal)		
No	179/168	1.00	200/206	1.00	
> 0 to ≤ 5.0	54/49	1.07 (0.65-1.76)	49/40	1.49 (0.84-2.64)	
> 5	28/29	0.92 (0.48-1.75)	34/19	2.31 (1.15-4.62)	
Per 5 g/day		0.98 (0.73-1.32)		1.51 (1.10-2.08)	
*P *trend		0.92		0.011	0.75

	≤ Median BMI (≤ 22.3 kg/m^2^)^d^		> Median BMI (> 22.3 kg/m^2^)		
No	155/172	1.00	224/202	1.00	
> 0 to ≤ 5.0	51/46	1.08 (0.64-1.85)	52/43	1.82 (1.06-3.12)	
> 5	36/22	2.36 (1.20-4.66)	26/26	0.99 (0.50-1.94)	
Per 5 g/day		1.42 (1.04-1.93)		1.15 (0.84-1.56)	
*P *trend		0.027		0.39	0.84

	Hormone therapy (HT) nonusers		HT users (former + current users)		
No	121/108	1.00	136/115	1.00	
> 0 to ≤ 5.0	31/17	1.97 (0.93-4.16)	24/11	2.28 (0.95-5.43)	
> 5	18/9	2.83 (1.04-7.67)	19/11	1.63 (0.68-3.94)	
Per 5 g/day		1.75 (1.11-2.76)		1.42 (0.93-2.15)	
*P *trend		0.016		0.10	0.34

^a^Adjusted for age, birthplace and residence years in the US among non-US born, education, interviewer, age at menarche, menopausal status, age at menopause, current BMI, parity, total calorie, family history of breast cancer and physical activity, intake of soy, and green tea. ^b^Low soy intake is defined as low intake during both adolescence and adult life or low intake during adolescence and high soy intake during adult life; high soy intake is defined as high soy intake during both adolescence and adult life or high intake during adolescence and low intake during adult life. ^c ^OSimilar patterns of results by tertiles of folate were found: ORs per 5 g/day of alcohol by tertiles of folate intake (≤ 170 mg/1,000 Kcal, > 170 to ≤ 220, > 220 mg/1,000 Kcal) were 1.09 (*P *trend = 0.65), 1.27 (*P *trend = 0.20), and 1.52 (*P *trend = 0.054), respectively. ^d^Similar patterns of results by tertiles of BMI were found: ORs per 5 g/day of alcohol intake by tertiles of BMI (≤ 21.5, > 21.5 to ≤ 24, > 24 kg/m^2^) were 1.34 (*P *trend = 0.09), 1.40 (*P *trend = 0.12), and 0.89 (*P *trend = 0.56), respectively.

Table 5 shows the lifetime alcohol/breast cancer association by estrogen receptor (ER) and progesterone receptor (PR) status among Japanese Americans. The alcohol/breast cancer associations (status, duration, and amount of drinking) were statistically significant among those with ER-positive (ER^+^) tumors but not among those with ER negative (ER^-^) tumors. Alcohol intake was positively associated with both PR^+ ^and PR^- ^breast cancer, although the findings were statistically significant only for those with PR^+ ^tumors. Further analysis by ER and PR status combined showed that increased risks were found among Japanese-American women with ER^+^/PR^+ ^tumors and those with ER^+^/PR^- ^tumors. The risk associated with 10^+ ^years of drinking was 1.56 (*P *trend = 0.034) for ER^+^/PR^+ ^tumors and 3.43 (*P *trend = 0.02) for ER^+^/PR^- ^tumors. No increased risk was found for those with ER^-^/PR^- ^tumors. The alcohol/breast cancer associations by hormone-receptor status in US-born Asian Americans (data not shown) were generally similar to the risk patterns observed in Japanese Americans.

**Table 5:** Lifetime alcohol intake and breast cancer risk^a ^in Japanese Americans by hormone-receptor status

	Estrogen receptor (ER)			Progesterone receptor (PR)			Estrogen (ER) and progesterone receptor (PR)				
	
	ER^+^	ER^-^	DK	PR^+^	PR^-^	DK	ER^+^PR^+^	ER^-^PR^-^	ER^+^PR^-^	ER/PR mix	ER/PR DK
Number	380	71	93	297	108	139	287	61	47	57	92
Any alcohol											
No	1.00	1.00	1.00	1.00	1.00	1.00	1.00	1.00	1.00	1.00	1.00
Former	1.19	1.56	0.91	1.15	1.64	0.94	1.17	1.98	1.47	0.86	0.92
Current	1.73^c^	0.66	1.42	1.61^c^	1.36	1.29	1.68^c^	0.84	2.76^c^	0.82	1.55
*P *(2 df)	0.008	0.62	0.39	0.028	0.21	0.49	0.017	0.88	0.035	0.68	0.27
Years of drinking											
No	1.00	1.00	1.00	1.00	1.00	1.00	1.00	1.00	1.00	1.00	1.00
> 0 to ≤ 10	1.31	1.32	1.37	1.07	2.25	1.20	1.35	1.73	1.20	0.74	1.38
> 10	1.67^c^	0.71	1.04	1.64	1.26	1.08	1.56^c^	0.87	3.43^c^	0.93	1.13
*P *trend	0.012	0.72	0.75	0.053	0.17	0.68	0.034	0.81	0.02	0.79	0.60
Amount of alcohol per day (g/day)											
No	1.00	1.00	1.00	1.00	1.00	1.00	1.00	1.00	1.00	1.00	1.00
> 0 to ≤ 5.0	1.41	1.12	0.99	1.28	1.61	1.04	1.33	1.38	2.22	1.01	1.03
> 5	1.65	0.84	1.60	1.68	1.26	1.35	1.72	1.12	2.06	0.60	1.70
*P *trend	0.019	0.90	0.34	0.034	024	0.46	0.023	0.56	0.09	0.53	0.26

E receptor positive (ER^+^), ER negative (ER^-^), ER unknown (ER DK); progesterone receptor positive (PR^+^), PR negative (PR^-^), PR unknown (PR DK). ^c^95% confidence intervals excluded 1.0.

Table 6 shows the effect of current alcohol intake on circulating hormones and other biomarkers in a subgroup of postmenopausal control women. After adjusting for age, Asian ethnicity, migration, education, age at menarche, parity, type of menopause, age at menopause, and BMI, levels of IGFBP-3 were 9% lower in current alcohol drinkers than in nondrinkers (*P *= 0.03), but differences in IGF-1 levels were not observed. Current alcohol consumers (average 6.3 g/day, 12.7 years of drinking) showed 10% to 16% higher levels of estrone and androgens (total testosterone, androstenedione, and DHEAS) than did nondrinkers, but these differences were not statistically significant. Current alcohol drinkers also showed borderline statistically significantly higher (17% to 19%) concentrations of SHBG (*P *= 0.09) and adiponectin (*P *= 0.10) than did nondrinkers.

**Table 6:** Adjusted^a ^mean concentrations of circulating hormones and other biomarkers^b ^by current alcohol intake among 196 postmenopausal control women

Alcohol^c^	E1 pg/ml	E2 pg/ml	Free E2 pg/ml	Total T ng/dl	Free T pg/ml	SHBG nM	Adione pg/ml	DHEAS μg/dl	IGF-1 ng/ml	IGFBP-3 μg/ml	Adiponectin ng/ml
No (*n *= 147)	31.3	10.2	4.1	17.9	5.2	38.8	461	64.0	115.7	4.4	12.0
Yes (*n *= 49)	34.7	10.3	5.0	19.6	6.5	45.5	509	74.4	113.8	4.0	14.3
*P*	0.11	0.92	0.32	0.31	0.27	0.09	0.20	0.23	0.78	0.03	0.10

^a^Least-square means were adjusted for age, Asian ethnicity, birthplace, education, age at menarche, parity, type of menopause, age at menopause, and current BMI. ^b^E1, estrone; E2, estradiol; Free E2, non-SHBG-bound estradiol; T, testosterone; Free T, non-SHBG-bound testosterone; SHBG, sex-hormone-binding globulin; Adione, androstenedione; DHEAS, dehydroepiandrosterone sulfate; IGF-1, insulin-like growth factor-1; IGFBP-3, insulin-like growth factor binding protein-3. ^c^Based on 49 current drinkers (11 Chinese, 18 Japanese, 20 Filipino) and 147 noncurrent drinkers (42 Chinese, 48 Japanese, 57 Filipino).

## Discussion

To our knowledge, this represents one of the most comprehensive analyses of lifetime alcohol intake and breast cancer risk in large numbers of Asian Americans that considered frequency, amount, duration, timing, and types of alcoholic beverages. Our results emphasize the heterogeneity of the Asian-American population and the need to study breast cancer risk factors separately by Asian ethnicity and by nativity. Regular lifetime alcohol intake is a significant risk factor in US-born Asian Americans but not in non-US-born Asian Americans. Our results by Asian ethnicity show that the prevalence of lifetime alcohol intake is significantly higher in Japanese than in Chinese and Filipino women (Table 1) and that lifetime alcohol intake is a significant risk factor in Japanese Americans (US born and non-US born) but not in Chinese- and Filipino-American women (Table 3, bottom). The risk estimates associated with amount and years of alcohol intake were very similar in US-born Japanese and non-US-born Japanese, but the results were statistically significant only in the former group, with more than twice the sample size as the latter group.

In a population-based case-control study that we and colleagues conducted in the 1980s, alcohol intake was unrelated to breast cancer risk in Chinese, Japanese, and Filipino Americans residing in California and Hawaii (OR was 0.9 (95% CI, 0.7 to 1.1) for ever drinking, defined as at least one drink per year) [2]. The percentage of weekly alcohol drinkers was higher in Japanese (21%) than in Chinese (13%) or Filipino (11%) control women, and a 40% increased risk was found among Japanese women who were weekly drinkers but not among Chinese or Filipino women. However, our previous study lacked information on duration and timing of alcohol intake.

In a cohort study conducted among multiethnic health-care enrollees at Kaiser Permanente, breast cancer risk in Asian Americans was significantly increased among regular alcohol consumers. Compared with Asians who were nondrinkers, the RRs among consumers of less than one drink per day, one to two drinks per day, and more than three drinks per day were 1.0 (95% CI, 0.6 to 1.4), 1.3 (95% CI, 0.8 to 2.4), and 3.5 (95% CI, 1.4 to 8.8), respectively, but this study also lacked information on the prevalence of drinking, other parameters of drinking, and the composition of Asians that were investigated [14].

Reasons for the significant effect of alcohol intake on breast cancer risk in Japanese Americans but not in Chinese and Filipino women are not apparent but may be related, in part, to the higher prevalence of alcohol intake among Japanese women in Asia and in the United States. Misclassification of alcohol intake cannot be ruled out, and this may be particularly relevant among non-US-born Asians, who were more likely to report increases in alcohol intake after migration (data not shown). Lifetime alcohol intake was unrelated to breast cancer risk for Chinese women in LAC, in agreement with findings from population-based case-control results from Shanghai, China [15,16]. Although alcohol intake was significantly inversely associated with risk in a hospital-based case-control study conducted among Chinese in Hangzhou, China (south of Shanghai) [17], alcohol intake among controls in that study (47%) was considerably higher than that in Shanghai (4%) [15,16], in Chinese in Singapore (5%) [18], and in those in Taiwan (7%) [19]. Lifetime alcohol was not a risk factor for LAC Filipino women, similar to the results from a case-control study conducted in the Philippines, where alcohol intake was also low (6.5% of breast cancer cases versus 8.5% of controls were drinkers) [20].

Results on alcohol intake and breast cancer risk in Japan are mixed with null findings in most of the hospital-based case-control studies (see review by Nagata *et al. *[3]). Although alcohol intake was unrelated to breast cancer risk in the Hiroshima [21] and the Miyagi [22] Cohorts, risk was significantly increased among heavy (> 15 g/day) drinkers (RR, 2.93; 95% CI, 1.55 to 5.54) in the Japanese Collaborative Study for Evaluation of Cancer Risk cohort study [23]. Similarly, in the Japan Public Health Center-based Prospective study, breast cancer risk was significantly increased among past drinkers (RR, 1.41; 95% CI, 1.09 to 1.83) and current heavy drinkers (> 23 g/day of alcohol) (RR, 1.76; 95% CI, 1.16 to 2.67) [24]. It is of note that the prevalence of alcohol intake appeared similar for contemporary Japanese women residing in the United States and for those in Japan. The prevalence of alcohol drinking among Japanese-American women in LAC (27% among US-born and 31% in non-US-born) is compatible with the intake of Japanese women in Hawaii (22%) [25] and with that of female participants in ongoing cohort studies in Japan (20% to 30%) [22-24].

All types of alcoholic beverages are implicated in our study. Most previous studies on alcohol intake and breast cancer risk conducted in Western populations did not find significant differences by type of alcoholic beverages (beer, wine, hard liquor) [14,26,27].

Although several risk factors (folate intake, hormone therapy use, BMI) have been implicated as potential modifiers of the alcohol/breast cancer association [22,28-30], these findings have not been consistently observed and were not significant modifiers in our study (Table 4). We found a significant effect of alcohol on breast cancer risk in Japanese Americans who were smokers but not among never smokers (Table 5). This finding differed from most previous studies conducted in women of European ancestry, which showed comparable effects of alcohol in smokers and never smokers [31]. Similar to the results reported in another Japanese study [24], we did not find any significant differences in the alcohol/breast cancer association by intake of soy.

In our study, the alcohol/breast cancer associations in Japanese Americans and US-born Asian Americans were statistically significant for women with ER^+^, PR^+^, and ER^+^PR^+ ^tumors, which are consistent with the results reported in other studies conducted in Western populations [4,32]. Interestingly, the effect of alcohol intake among Japanese Americans with ER^+^/PR^- ^was particularly prominent and is supportive of findings reported in the Nurses' Health Study and a study in Shanghai, China [4,16].

A favored hypothesis is that alcohol intake is associated with increases in sex hormone concentrations, which might contribute to the increase in breast cancer risk [33]. Our cross-sectional results among postmenopausal Asian control women suggest elevated concentrations of estrone and androgens (total testosterone, androstenedione, and DHEAS) and are qualitatively similar to the results reported by the Endogenous Hormones and Breast Cancer Collaborate Group, which pooled cross-sectional data from more than 6,000 postmenopausal women, almost all of whom were of European ancestry [34]. However, we found higher SHBG levels among alcohol consumers, whereas their results showed lower SHBG levels [34]. Our findings of borderline significantly higher adiponectin levels in alcohol drinkers than in nondrinkers are supportive of results from cross-sectional [35,36] and intervention studies [37,38]. In our study, IGF-1 levels did not differ by alcohol intake in Asian-American women, but the levels of IGFBP-3 were significantly lower in current consumers. Similarly, most previous studies in postmenopausal women have found little effect of alcohol on IGF-1 levels [36,39,40]. The effect of alcohol intake on IGFBP-3 levels was not consistent in previous studies; no significant changes [40] or significantly lower [36] and higher levels [39] were reported previously.

Notwithstanding this, several limitations should be noted. The overall participation rate was modest (61% among cases and 64% among controls). Although our refusal rate (23%) is comparable to rates reported in other US studies, some 14% of the identified cases had moved outside of LAC (21% for Filipino, 12% for Chinese, and 8% for Japanese). A methodologic concern is the comparability of cases we interviewed and those we did not interview in terms of alcohol intake. Nevertheless, we have reasonable confidence in our findings among the respondents because we have two separate measures of alcohol intake, and the results were similar. One measure was based on responses obtained from the FFQ, and the intake of beer, wine, and hard liquor was asked along with the other more than 100 food items/groups included in our questionnaire. A second measure was based on lifetime history assessment of alcohol intake.

Misclassification of exposure is another concern in this and other case-control studies. However, our findings on alcohol intake by hormone-receptor status are generally compatible with published results from cohort studies [4,32]. This is reassuring, because case respondents typically are not aware of the hormone-receptor status of their breast tumors.

Finally, although the sample size of our cross-sectional study among control women was modest, our findings of elevated estrone, androgen, and adiponectin levels among current alcohol consumers compared with nonconsumers are also compatible with larger studies conducted in Caucasian women [34].

## Conclusions

Alcohol-drinking habits differ by Asian ethnicity and nativity, and their effects on breast cancer risk differ accordingly. Analyses by nativity and Asian ethnicity revealed a statistically significant alcohol/breast cancer association in US-born Asian Americans and Japanese Americans, emphasizing the growing importance of this risk factor in traditionally low-risk populations as alcohol intake has become more prevalent. Because alcohol intake is one of the few modifiable breast cancer risk factors, Asians and other traditionally low-risk populations should be aware of the risk associated with even moderate amounts of intake. Further studies are also needed to elucidate the mechanisms of the effect of alcohol intake on breast cancer risk in Asians and in the non-Asian populations.

## Abbreviations

Adione: androstenedione; Adj: adjusted; ANOVA: analysis of variance; BMI: body mass index; CI: confidence interval; CSP: Cancer Surveillance Program; DHEAS: dehydroepiandrosterone sulfate; E1: estrone; E2: estradiol; ER: estrogen receptor; FFQ: food-frequency questionnaire; IGF-1: insulin-like growth factor-1; IGFBP-3: insulin-like growth factor-binding protein 3; LAC: Los Angeles County; OR: odds ratio; PR: progesterone receptor; SHBG: sex-hormone-binding globulin; SEER: Surveillance, Epidemiology, and End Results; T: testosterone.

## Competing interests

The authors declare that they have no competing interests.

## Authors' contributions

AW was responsible for the design and implementation of study and supervised the data-collection activities. Data management was conducted by CT. CT, CV, and PR conducted data analyses under the supervision of AW. Laboratory measurements were conducted under the supervision of FS. All authors read and approved the manuscript.
